# Educational Inequalities in Women’s Depressive Symptoms: The Mediating Role of Perceived Neighbourhood Characteristics 

**DOI:** 10.3390/ijerph9124241

**Published:** 2012-11-22

**Authors:** Megan Teychenne, Kylie Ball, Jo Salmon

**Affiliations:** Centre for Physical Activity and Nutrition Research, School of Exercise and Nutrition Sciences, Deakin University, 221 Burwood Hwy, Burwood, VIC, 3125, Australia; Email: kball@deakin.edu.au (K.B.); jo.salmon@deakin.edu.au (J.S.)

**Keywords:** socioeconomic position, depression, women, neighbourhood

## Abstract

Socio-economically disadvantaged (e.g., less educated) women are at a greater risk of depression compared to less disadvantaged women. However, little is known regarding the factors that may explain socioeconomic inequalities in risk of depression. This study aimed to investigate the contribution of perceived neighbourhood factors in mediating the relationship between education and women’s risk of depression. Cross-sectional data were provided by 4,065 women (aged 18–45). Women self-reported their education level, depressive symptoms (CES-D 10), as well as four neighbourhood factors (*i.e.*, interpersonal trust, social cohesion, neighbourhood safety, and aesthetics). Single and multiple mediating analyses were conducted. Clustering by neighbourhood of residence was adjusted by using a robust estimator of variance. Multiple mediating analyses revealed that interpersonal trust was the only neighbourhood characteristic found to partly explain the educational inequalities in women’s depressive symptoms. Social cohesion, neighbourhood aesthetics and safety were not found to mediate this relationship. Acknowledging the cross-sectional nature of this study, findings suggest that strategies to promote interpersonal trust within socioeconomically disadvantaged neighbourhoods may help to reduce the educational inequalities in risk of depression amongst women. Further longitudinal and intervention studies are needed to confirm these findings.

## 1. Introduction

Socioeconomic disadvantage is linked to poor health outcomes, such that adults of a low socioeconomic position are at a greater risk of obesity [1], cardiovascular disease [2] and mortality [3,4]. Further, it is now well-established that socioeconomic position is inversely associated with risk of depression [5], with studies from developed countries including the US [6], UK [7] and Australia [8] indicating that disadvantaged adults are an at-risk group of experiencing depression, regardless of the indicator used to measure socioeconomic disadvantage (e.g., education, income, occupation). Despite the considerable number of studies demonstrating socioeconomic inequalities in depression [5], very few have aimed to explore the underlying factors (*i.e.*, mediators) which may explain this relationship. Of the limited research that has assessed potential mediators of the socioeconomic position-mental health gradient, intra-personal factors have generally been assessed. For example, previous research has indicated that factors such as health behaviours (e.g., smoking, alcohol consumption, physical inactivity) [9,10,11], somatic health (e.g., CVD, overweight/obesity, physical function) [9,11], work-related factors (e.g., job satisfaction, tiredness due to job) [11], psychosocial factors (e.g., support network size, self-efficacy) [9] and sociodemographic factors (e.g., marital status) [11] partly mediated the relationship between socioeconomic position and depressive symptoms. Results from these studies showed that socioeconomic inequalities in risk of depression remained largely unexplained, underscoring the need for further investigation of these pathways, particularly among factors other than those at the intra-personal level [9,12]. 

It has been suggested that the link between socioeconomic position and physical health may be mediated by characteristics related to social capital, such as social trust (*i.e.*, level of trust in neighbours/politicians/people in general [13]) and social cohesion (*i.e.*, an absence of conflict within society, as well as sharing common values [14]) [15,16,17,18]. For example, the cross-sectional study by Kawachi *et al.* [18] found that the inverse relationship between socioeconomic position and mortality was partly explained by social capital factors including social trust and group membership (*i.e.*, civic involvement). Further, a small body of research has found a positive association between social capital (e.g., social trust, social participation and neighbourhood cohesion) and mental health [19,20,21,22]. For example, Lindstrom *et al.* [20] concluded that Swedish adults reporting low social participation and social trust were at increased risk of poor psychological health. 

Neighbourhood socioeconomic disadvantage has been shown to be correlated with health outcomes such as CVD [23,24] and functional status [24]. Furthermore, a number of studies have explored the relationship between the built neighbourhood environment and mental health with a large proportion of studies showing an association between the two factors [25,26]. For example, the review by Mair *et al.* [26] concluded that all four studies that assessed the relationship between the built environment and depressive symptoms found that the prevalence of depression was associated with characteristics of the neighbourhood environment, which included the quality of housing, walking environment, violence and abandoned buildings [27,28,29,30]. Further, research has suggested that living in unsafe neighbourhoods [22] and perceiving neighbourhood problems (e.g., traffic density, pollution, absence of local facilities, limited public transport) [31] may also be linked to psychological distress. 

Very little is known as to whether neighbourhood characteristics mediate the relationship between socioeconomic position and mental health. One study providing some insight into this relationship utilised concept mapping techniques to examine the influence of socioeconomic position on perceptions of the relationship between the neighbourhood and mental well-being [32]. That study found that perceptions of the role of the neighbourhood on mental health varied by individual’s socioeconomic position (e.g., disadvantaged adults felt that good public and social services were important for mental health, however less disadvantaged adults felt those factors were not important). The authors concluded that more quantitative and multi-level research was needed to better elucidate the neighbourhood characteristics that explain socioeconomic inequalities in mental health [32].

Women are at greater risk of experiencing depression compared to men [8], with nearly 20 percent of Australian women suffering from depression in their lifetime [33]. Therefore, this study aimed to investigate the perceived neighbourhood characteristics that mediate the relationship between education and risk of depression in women. Understanding the perceived neighbourhood factors (social and physical) that explain educational inequalities in depression may provide insights for the development of interventions, policies and urban planning practices aimed to reduce risk of depression in disadvantaged populations. 

## 2. Methods

Analyses were based on cross-sectional survey data collected from the Resilience for Eating and Activity Despite Inequality (READI) Study of 2007/2008. Data used in analyses were provided by 4,065 women (aged 18–45) residing in socio-economically disadvantaged neighbourhoods in Victoria, Australia. Methods have been described in detail elsewhere [34], thus are briefly outlined below. 

### 2.1. Participants

Participants were recruited from 80 Victorian neighbourhoods (suburbs) (40 rural and 40 urban (*i.e.*, encompassing metropolitan cities and surrounds)) of low socio-economic position, based on the Australian Bureau of Statistics SEIFA—Socioeconomic Index for Areas, which is the most widely-used measure of area level SEP in Australia, and is based on aggregated variables including the proportions of residents with particular housing status, occupation, income and level of completed education [35]. The electoral roll was then used to randomly select approximately 150 women from each of the 80 suburbs, aged between 18 and 45 years. 

Surveys were sent to a random sample of 11,940 women, and a total of 4,934 women returned a completed survey, representing a response rate of 45% [34]. Compared with respondents, non-respondents were more likely to live in urban than rural areas, and lived in neighbourhoods with lower mean SEIFA scores (representing greater area-level disadvantage). Of the respondents, 571 women were excluded due to residing in neighbourhoods outside of those selected for the study. Another 9 women were excluded due to falling outside the valid age range (*i.e.*, either younger than 18 years or older than 46 years, or had data missing on this variable). Three women were excluded since the survey was not completed by the woman it was addressed to and two women later withdrew from the study. This left a total of 4,349 women included in the overall study. Since pregnancy is likely to affect depressive symptoms [36], a further 284 women (6%) were excluded from analyses because they reported being pregnant, didn’t know their pregnancy status, or did not complete this question. This left a total of 4,065 women (82% of the original respondents) with data for inclusion in the analyses. 

### 2.2. Procedures

The study was approved by the Deakin University Human Research Ethics Committee. Surveys were posted out one week after initially sending women a pre-survey letter in the mail, informing them that they had been selected to take part in a study on women’s health. Following the Dilman protocol [37], non-respondents received a mailed reminder 10 days later and a second reminder with a replacement survey a further 10 days later. Women received small incentives (e.g., tea bags, $1 scratch lottery tickets) with their initial survey package. Written consent to participate was obtained from all respondents. 

### 2.3. Measures

#### 2.3.1. Explanatory Variable

Highest educational level was used as an indicator of socio-economic position. This was categorised as either “Low: no formal qualifications/up to year 10”, “Medium: year 12/trade/apprenticeship/certificate/diploma”, or “High: university degree/higher degree”. It has been argued that education level is a suitable proxy for socioeconomic position for women, since it reduces the incidence of issues relating to the instability of markers such as occupation and income that often change for women who move in and out of the workforce during childrearing years [38]. 

#### 2.3.2. Outcome Variable—Depressive Symptoms

Depressive symptoms were assessed using the 10-item version of the Centre for Epidemiologic Studies Depression Scale (CES-D), a reliable and well-validated measure of depression [39,40]. It includes questions that relate to various symptoms of depression that may have been experienced in the past week, which indicate whether a participant is at risk of depression. Respondents rated themselves on a 4-point severity scale. Scores were summed and analysed as a continuous variable. For descriptive purposes CES-D scores of 10 or greater indicated that the participant was at risk of depression [40,41,42]. 

#### 2.3.3. Mediating Variables

Four potential mediators were assessed in this study. Interpersonal trust was assessed using two items which asked participants to rate on a 5-point Likert scale how strongly they agreed (1 = strongly disagree to 5 = strongly agree) with the statements “Most people can be trusted” and “Most of the time people try to be helpful” [43] (ICC = 0.75). Social cohesion within the community was assessed using a five-item measure which asked participants to rate on a 5-point Likert scale how strongly they agreed (1 = strongly disagree to 5 = strongly agree) with five statements (e.g., “People in the neighbourhood can be trusted”; “People around here are willing to help their neighbours”) [44] (ICC = 0.85). Perceived neighbourhood aesthetics were measured with five items [45]. Participants were asked to indicate on a five-point Likert scale how strongly they agreed (1 = strongly disagree to 5 = strongly agree) with five statements (e.g., “my neighbourhood is attractive”; “in my neighbourhood the buildings and homes are well-maintained”) (Cronbach’s = 0.76; Kappa range = 0.45–0.64). Perceived safety in the neighbourhood was assessed with three items (Cronbach’s = 0.85; Kappa range = 0.42–0.43). Participants were asked to indicate on a five-point Likert scale how strongly they agreed (1 = strongly disagree to 5 = strongly agree) with three statements (e.g., “I feel safe walking in my neighbourhood, day or night”; “my neighbourhood is safe from crime; “violence is not a problem in my neighbourhood”). Scores were each summed then analysed as continuous variables. 

### 2.4. Covariates

Self-reported marital status, BMI, age, employment status and leisure-time physical activity were included in single and multiple mediating analyses as potentially confounding factors, since these were bivariately associated with depressive symptoms (*i.e.*, being married, of normal weight, working full-time, of younger age and reporting higher levels of leisure-time physical activity were associated with lower levels of depressive symptoms). The urban/rural status of the neighbourhood was not associated with depressive symptoms and thus not included as a covariate.

### 2.5. Statistical Analyses

Analyses were performed using STATA version 11.0. Descriptive and unilevel analyses were used to examine the distributions of, and bivariate associations between, depressive symptoms, demographic, education and mediator variables. MacKinnon’s product of coefficients test of statistical mediation was used since it has been argued to provide greater statistical power than other commonly-used mediating methods [46]. After testing the distributions of outcome variables for normality (and then transforming these to be as close as possible to a normal distribution using either a square root or log transformation), a linear regression model (*i.e.*, single mediating analysis) was used to bivariately estimate the contribution of environmental mediators to explaining educational variations in women’s depressive symptoms. Clustering by neighbourhood of residence was adjusted by using a robust estimator of variance. This was performed by following MacKinnon’s product of coefficients formula (z = αβ/SEαβ), whereby α = the relationship between the independent variable (education) and the mediator; β = the relationship between the mediator and the dependent variable (depressive symptoms), adjusting for the independent variable; and SEαβ = the standard error of both α and β [46]. A z-score greater than the absolute value of 1.96 (*i.e.*, greater than 1.96 or less than −1.96) was used to indicate a statistically significant mediating association. Following this, a multiple mediation analysis was performed, and only the proposed mediators that were found to be significantly associated with depressive symptoms in single mediating analyses were included in the multiple mediation model. The standard error was calculated using the Sobel [47] method, which is that most commonly used [46] SEαβ = (SQRT((α^2^ × SEβ^2^) + ( β^2^ × SEα^2^)). 

## 3. Results

Table 1 presents the socio-demographic characteristics of participants. The mean age of participants was 35 years (SD = 8.22) and just under a quarter of women (23%) reported not completing high school. A total of 1,540 women (38%) reported being at risk of depression indicating a CES-D score of 10 or greater. 

**Table 1:** Socio-demographic characteristics of participants (n = 4,065).

Characteristic	N	Percent
*Highest Qualification*		
Did not complete high school	910	23
High school/trade apprentice/Certificate diploma	2,062	52
University or Higher degree	1,030	25
*Age*		
Under 25 years	698	17
25 to 29 years	549	14
30 to 34 years	597	15
35–39 years	849	21
40+ years	1,318	33
*Marital Status*		
Married or defacto	2,597	64
Separated widowed or divorced	359	9
Never married	1,083	27
*Employment status*		
Working full-time	1,521	38
Working part-time	1,170	30
Not currently employed in paid work	1,265	32
*BMI*		
Not overweight (<25)	2,057	54
Overweight (≥25–30)	964	25
Obese (≥30)	826	21
*Leisure-time physical activity*		
Low (<40 min/week)	1,293	33
Moderate (40min–3.4 h/week)	1,262	32
High (≥3.4 h/wk)	1,357	34.7
*Depressive symptoms*		
Not at risk (<10)	2,525	62
At risk (≥10)	1,540	38

Education was inversely associated with women’s depressive symptoms (regression coefficient (τ) = −0.12; 95%CI = −0.20, −0.05). Table 2 presents the bivariable and multivariable associations between education, and neighbourhood factors hypothesised to mediate the relationship between education and depressive symptoms. The bivariable results showed that interpersonal trust and neighbourhood aesthetics were significant mediators of educational variations in depressive symptoms, explaining 33% and 34% of the relationship between education and depressive symptoms respectively. However, social cohesion and neighbourhood safety were not found to be mediators of educational variations in depressive symptoms. In the multivariable model, only interpersonal trust remained a significant mediator of educational variations in depressive symptoms, explaining 33% of the relationship between education and depressive symptoms. 

**Table 2:** Potential mediators from single and multiple mediating analyses explaining the association between education and depressive symptoms amongst women.

Mediators	α (95% CI)	β (95% CI)	αβ	SEαβ	z-score
*Single mediating analyses*					
Interpersonal trust	2.58 (1.12, 4.04)	−0.01 (−0.02, −0.01)	−0.026	0.01	−2.58 *
Social cohesion	3.81 (−4.47, 12.09)	−0.00 (0.00, 0.00)	−0.01	0.01	−0.88
Neighbourhood aesthetics	12.1 (3.22, 20.98)	−0.00 (−0.00, −0.00)	−0.021	0.01	−2.06 *
Neighbourhood safety	−0.24 (−0.31, 0.26)	−0.05 (−0.06, −0.04)	0.001	0.01	−0.11
*Multiple mediating analyses*					
Interpersonal trust	2.58 (1.12, 4.04)	−0.01 (−0.01, −0.01)	−0.033	0.01	−3.3 *
Neighbourhood aesthetics	12.1 (3.22, 20.98)	−0.00 (−0.00, −0.00)	−0.015	0.01	−1.45

* *p* < 0.05. Adjusted for marital status, BMI, employment status, age, leisure-time physical activity and clustering by neighbourhood.

## 4. Discussion

Although studies have examined correlates of mental health, most have investigated psychosocial correlates (e.g., self-efficacy) [9]. Thus, little is known in regards to the neighbourhood characteristics that may be associated with depressive symptoms, and in particular, whether those characteristics may explain socioeconomic inequalities in risk of depression. Hence, this is one of the first studies to examine the role of neighbourhood characteristics in explaining educational inequalities in women’s depressive symptoms. 

In this study, education was inversely associated with depressive symptoms, which parallels findings of previous literature [5], and further highlights the need to understand the underlying factors that may explain the socioeconomic inequalities in mental health. Of the factors examined in this study, interpersonal trust was the only characteristic of the neighbourhood that was found to partly mediate the relationship between education and women’s depressive symptoms. Consistent with previous research [48], women who reported high levels of education also indicated greater perceived levels of interpersonal trust than did women who reported low levels of education. One possible explanation for this is that individuals with lower levels of education may not have the social and/or economic resources that encourage trust [48,49]. For example, having a small support network or few valuable assets may lead to a sense of defensiveness in order to protect what one does have [48]. Further, women with low levels of education may be more likely to live in disadvantaged neighbourhoods, which may be characterised by higher levels of crime and thus lower levels of neighbourhood trust [50,51]. 

This study demonstrated an inverse association between interpersonal trust and depressive symptoms, which is consistent with previous findings [20,22] and indicates that having greater trust in others may play an important role in reducing depressive symptoms in women. It may be that women find it distressing living in a community where there is a low level of trust which may result in an increase in depressive symptoms. Moreover, it has been suggested that residing in a more trusting community may benefit those with depression as people living in those neighbourhoods may have better access to support services [52,53]. Therefore, improving social capital, in particular promoting trust in the community may be a key approach to reduce risk of depression, particularly amongst less educated women, a group who are already at an increased risk of depression [5,8]. 

Alternatively, given the cross-sectional nature of this study, the inverse relationship between interpersonal trust and depressive symptoms may operate in the reverse direction. That is, experiencing depressive symptoms may lead to feelings of mistrust in others. Phongsavan *et al.* [22] suggested that mental health issues can influence individual’s perceptions of their social environment (*i.e.*, social trust), thus low interpersonal trust may reflect underlying mental health problems rather than the social environment influencing an individual’s mental health. However, findings from one previous study that found an inverse relationship between social trust and depression [52], indicated that based on their longitudinal study design this explanation (*i.e.*, mental illness lead to perceptions of mistrust) was unlikely. Rather, the authors suggested that living in a community with greater trust may have a protective effect against depression. An alternative scenario is that a third unmeasured variable, such as a history of abuse or other adverse life circumstances, may explain the risk for low socioeconomic position [54], low trust in others [55] and depression [56]. Since little is known about the causality of this relationship, the need for further studies—particularly qualitative and longitudinal studies, to help tease out the underlying mechanisms—is underscored. 

Given that women of a low education may be more likely to live in neighbourhoods of low socio-economic position (e.g., with higher levels of crime, lower levels of neighbourhood trust), strategies to increase interpersonal trust within disadvantaged neighbourhoods are needed. In addition to structural approaches to reducing crime, incivilities and other adverse neighbourhood factors, implementing social activities (e.g., walking groups) and developing local support groups that promote trust within the neighbourhood may be important strategies that may help reduce the risk of depression amongst less educated women. 

As already highlighted, a major limitation of this study is the cross-sectional study. However, since little research has investigated the role neighbourhood characteristics in mediating socio-economic gradient in women’s depressive symptoms, the present cross-sectional findings provide important initial insights in this research area. Secondly, self-report measures were used to assess depressive symptoms as well as potential mediating factors, which may be subject to error in judgment and socially desirable responses. Since it has been established from physical activity research that the perceived and objective environment is not strongly correlated [57] (*i.e.*, perceptions of environment are often more important predictors of physical activity than the actual environment), it may be important to determine which measures of the environment (perceived or objective) are more important for predicting depression. Thus, future studies could utilise objective measures of the neighbourhood environment such as neighbourhood audits or crime statistics. Finally, socioeconomic position was defined using the individual-level measure, education. Although this is a measure that has been widely used to indicate socio-economic position in other epidemiologic and health-related studies amongst women [58,59], a number of participants classified as having low education may not have been considered to be socio-economically disadvantaged based on other indicators such as income or occupation. However, education is a useful measure of socio-economic position amongst women, as it remains relatively stable during adult life in contrast to income and occupation, which fluctuate particularly during childbearing years [38]. 

A major strength of this study is the use of a large, population-based sample of women; this provided good power to detect associations, even after controlling for a range of important covariates. Since literature on the neighbourhood characteristics that may explain the relationship between education and depressive symptoms amongst adult women is particularly scarce, the findings from the current study provide novel information which, if confirmed in future studies, may be used to inform intervention strategies to reduce educational inequalities in women’s risk of depression. 

## 5. Conclusions

Since depression is now the leading cause of disease burden amongst women living in both low-medium and high income countries [60], it is imperative that strategies to prevent and manage women’s depressive symptoms are identified. Acknowledging the cross-sectional design, this study suggests that strategies to further promote interpersonal trust within socioeconomically disadvantaged neighbourhoods such as establishing recreational clubs or support groups/services may be important in order to reduce the educational inequalities in risk of depression amongst women. Although findings need to be confirmed with further longitudinal, qualitative and intervention studies, this study provides novel insights for informing the development of interventions and policies aimed to prevent and/or manage depression in less educated populations. 
